# Awareness of Anterior Cruciate Ligament Injury—Preventive Training Programs among Saudi Athletes

**DOI:** 10.3390/clinpract13030060

**Published:** 2023-05-29

**Authors:** Ali H. Alyami, Hussam Darraj, Sulaiman Hamdi, Abdulaziz Saber, Nawaf Bakri, Rawan Maghrabi, Khalid M. Hakami, Anwar Darraj

**Affiliations:** 1 Department of Surgery, Ministry of the National Guard Health Affairs, Jeddah 21423, Saudi Arabia; 2 Department of Surgery, King Abdullah International Medical Research Center, Jeddah 21423, Saudi Arabia; 3 Department of Surgery, King Saud bin Abdulaziz University for Health Sciences, Jeddah 21423, Saudi Arabia; 4 Faculty of Medicine, Jazan University, Jazan 45142, Saudi Arabia; 5 College of Medicine, King Saud bin Abdulaziz University for Health Sciences, Jeddah 21423, Saudi Arabia

**Keywords:** preventive training programs, ACL injury, athletes, knee, neuromuscular training

## Abstract

Background: Anterior cruciate ligament (ACL) tear is a common medical condition that entails a stretch or sprain of the ACL, which is present in the knee joint. The incidence of ACL injury in the Kingdom of Saudi Arabia is estimated to be 31.4%. Prevention training programs (PTPs) can be used to reduce ACL injuries sustained during physical activity, as they primarily focus on improving strength, balance, and lower limb biomechanics and reducing landing impact. This study aimed to assess Saudi athletes’ awareness of ACL injury PTPs. Methods: A cross-sectional survey in the form of a self-administered questionnaire in the Arabic language was carried out from 22 December 2022 to 7 March 2023 and included 1169 Saudi athletes. Statistical analyses were performed on the collected data using frequency and percentages. Binary logistic regression was used for the adjusted analysis and determining associations between athletes playing high- and low-risk sports. Results: Overall, 52% of participants were female athletes, and 48% were male athletes. The western region of the country had the highest response rate (28.9%). The most common sport played was football at 36.6%. Most participants (70.97%) reported that their information on ACL injury was taken by their coaches. When assessing whether participants were familiar with the concept of an ACL injury PTP, the majority of the participants answered no, representing 971 (662 high-risk, 309 low-risk), compared to those who answered yes, representing only 198 (167 high-risk, 31 low-risk), with a statistically significant difference (adjusted OR: 2.106; 95% confidence interval: 1.544–2.873; *p*-value < 0.001). Conclusion: In general, the level of awareness of ACL injury PTPs among Saudi athletes was poor.

## 1. Introduction

The human knee joint is a complex structure designed to function as a pivot point for moving and bearing body weight; thus, it is often subject to injury. As a connective structure in the human leg, it joins three bones, the tibia, femur, and patella, with four connecting ligaments, namely the medial collateral ligament, lateral collateral ligament, posterior cruciate ligament, and anterior cruciate ligament (ACL). Among these, the ACL has a primary stabilizing role in the knee joint, as it connects the tibia and femur, restraining the tibia’s internal rotation and translation [1,2].

An anterior cruciate ligament tear is a common medical condition that entails a stretch or sprain of the ligament [1,2]. In the United States, the percentage of ACL injuries reached 200,000 needing repair, and the rate of ACL injury in Germany was 32 injuries per 100,000 people. In addition, the incidence of ACL injury in the Kingdom of Saudi Arabia has been estimated at 31.4% according to a study conducted in Riyadh [3]. Such injuries are frequently encountered due to a sudden intense shock during knee movement. Two types of injuries can cause ACL tears, namely contact injuries involving direct collision of the knee with a solid object and non-contact injuries involving intense forceful exertion of the knee due to an imbalanced movement or a fault in a technical movement. Non-contact ACL injuries are considered to be more common, accounting for almost 70% of ACL tears. These injuries may arise due to factors, including a sudden change in the speed or direction of movement, pivoting, or awkward landing [4,5].

A torn ACL usually occurs as a result of an acute non-contact deceleration injury, forceful hyperextension, or excessive rotational forces about the knee [6,7]. The ligament may be completely torn, partially torn, or avulsed from its origin or insertion. The ACL is the primary restraint to excessive anterior translation and rotation of the tibia on the femur; therefore, complete ACL disruption typically results in dynamic knee instability or the inability to respond to quick changes in position [8].

Anterior cruciate ligament injuries are a common problem in female athletes, who are two- to nine-times more likely to sustain such injuries than their male counterparts [9,10,11]. The prevalence of ACL reconstructions in women continues to rise [12,13]. The annual cost of ACL surgeries is estimated at USD15 million. The cost per surgery ranges from USD800 to USD3000. This cost includes hospital admission fees, post-surgical visits to the doctor, and other related expenses [14]. Someone with an ACL injury will be prevented from practicing sports until an operation and comprehensive rehabilitation are performed; they may be allowed to exercise after approximately six months [15].

Preventive training programs (PTPs) can be used to reduce ACL injuries sustained during physical activity, as they primarily focus on improving strength, balance, and lower limb biomechanics and minimizing landing impact [16]. However, the implementation of these cost-effective programs seems to vary based on factors, such as age, gender, sport, awareness, compliance, and program type.

There is a gap in the knowledge regarding PTPs for ACL injury in Saudi Arabia. Therefore, the present study aimed to evaluate awareness of this topic among Saudi athletes. The data obtained will aid in the understanding of the impact of ACL injuries on various aspects of athletes’ lives.

## 2. Subjects, Methods, and Materials

### 2.1. Study Design, Setting, and Population

This cross-sectional observational study focused on assessing the awareness of ACL injury PTPs among Saudi athletes. It was carried out in Saudi Arabia, which has a population of 35 million and is situated in the far southwest of the continent of Asia. It is bordered on the west by the Red Sea, on the east by the Arabian Gulf, the United Arab Emirates, and Qatar, on the north by Kuwait, Iraq, and Jordan, and on the south by Yemen and the Sultanate of Oman. The study targeted all athletic adults in Saudi Arabia who met our inclusion criteria: all athletic males and females aged ≥18 years who lived in different regions of Saudi Arabia at the time of the study and completed the survey. Those who refused to participate were excluded from the study.

### 2.2. Data Collection

The survey was conducted from 22 December 2022 to 7 March 2023, in all regions of Saudi Arabia in the form of a self-administered validated questionnaire obtained from a previously published study [16]. The data were collected using the Google platform to determine Saudi athletes’ knowledge and awareness of ACL injury PTPs. The Google Form link was posted on and circulated among various social media platforms, such as WhatsApp groups, Facebook, Instagram, and emails.

The survey took about 4–5 min to complete. The survey contained three sections, as follows: first section to assess the sociodemographic characteristics, including age, gender, nationality, place of residence (north, west, east, and south regions of Saudi Arabia), and type of sport that they practice. The second and third sections included a 12-item survey to assess the participants’ awareness about ACL injury and PTP experience, as described in Table 1. True and false questions were used to assess the participants’ beliefs regarding whether the prevalence of ACL injury is more common in females compared to males, whether ACL injuries could be prevented, and their knowledge, awareness, and experiences of ACL PTP programs.

Respondents’ interest levels in carrying out a daily PTP were evaluated using a Likert scale consisting of 7 points, where a score of 1 indicated no interest and a score of 7 indicated a high level of interest.

Each question aimed to evaluate a specific component of a specific research goal. Using the sample size formula for studies with cross-sectional study designs, a total of 385 participants were determined to be the appropriate sample size for this investigation. The study used *p* = 50% to determine the maximum sample size, the 95% confidence interval (CI), and an error of no more than 5%. Additionally, a 25% non-response rate was anticipated for the survey. The simple random sampling technique was utilized for the sample design.

### 2.3. Pilot Study

Before conducting the full study, a pilot study was carried out with 10% of the required sample size to assess the participants’ understanding and clarity of the questionnaire used for data collection. Certain improvements and reordering of some questions were made based on the results of the pilot study. The results of the pilot study were not included in the analysis of the final data.

### 2.4. Statistical Analysis

The data were collected in an Excel sheet and then checked and cleaned for incomplete information and incorrect values. Descriptive data were used to present the information. Categorical variables were presented as frequencies and percentages. Binary logistic regression was used for the adjusted analysis and determining association athletes playing high- and low-risk sports. The analysis was performed using the IBM SPSS software (version 25) (Raosoft Inc., Seattle, WA, USA).

### 2.5. Ethical Considerations

The study protocol was approved by the internal review board of the Ethics Committee of King Abdullah International Medical Research Center (approval no.: NRJ22J/231/09; date 22 December 2022). Informed consent to participate was obtained from each participant before they filled out the online questionnaire. Participation was completely voluntary, and participants could withdraw from the study if they wished.

The total number of participants who responded to the electronic survey was 1169. The majority of participants were females, representing (52%). The mean age was 30.99 years (SD = 11.25). The western region had the highest response rate (28.9%), followed by the central region (21%). The most common sport played was football (36.6%), followed by cardio (21.8%), athletics (10.7%), and water sports (9.3%), as described in Table 2.

Based on the data analyzed, when the female athletes’ association athletes were asked whether they knew what an ACL injury was, 63.1% answered yes. When asked whether they believed that female athletes are at increased risk of sustaining ACL injuries, 19.8% said yes, 20.4% answered no, and 59.9% said they did not know. Concerning whether female and male athletes should receive the same treatment for the same injuries, 40.9% agreed, 14.7% disagreed, and 44.4% said they did not know. When asked whether they believed that ACL injuries are preventable, 67.2% said yes, 3.4% said no, and 29.4% said they did not know. Finally, 37.1% said they had been educated on ACL injury, and 62.0% said they had not been. In addition, the majority of the participants had a great interest in learning more about ACL preventive training programs, with a percentage of 36.4%, followed by those who had a slight interest, with a percentage of 17.4% (Table 3).

**Figure 1:**
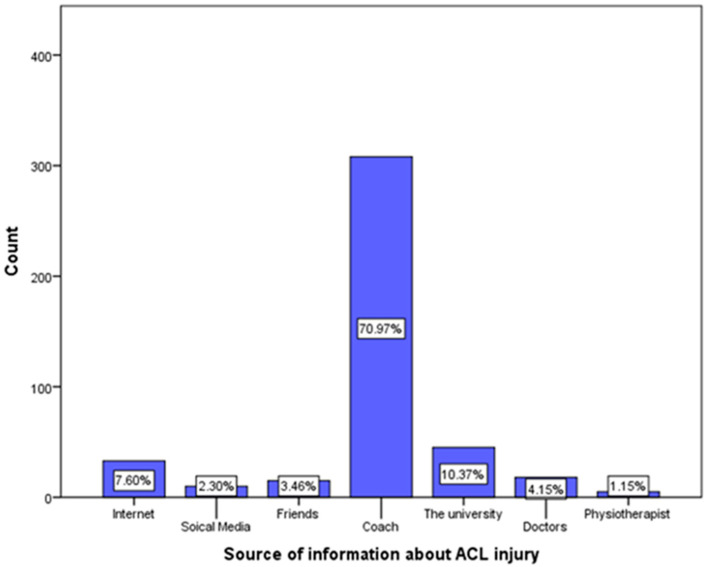
Source of information about anterior cruciate ligament injury.

As shown in Figure 1, the most common information source on ACL injury was coaches (70.97%), followed by the athlete’s university (10.37%), the internet (7.60%), and doctors (4.15%).

**Figure 2:**
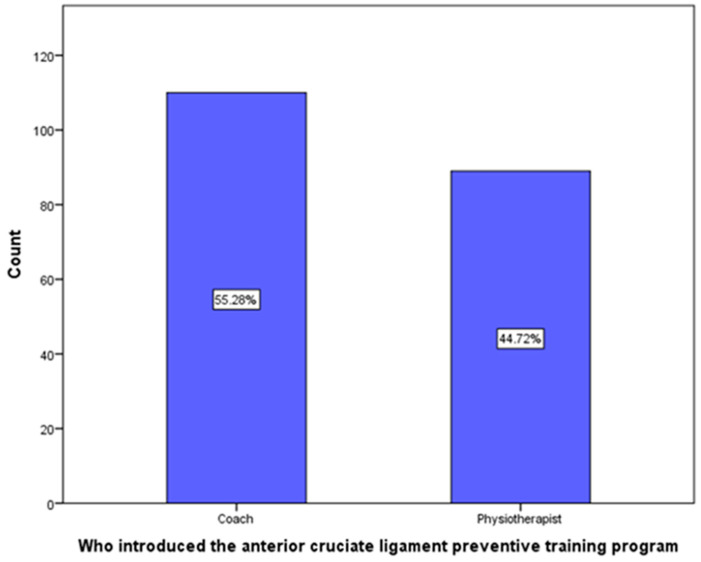
Distribution of educators providing training to survey respondents who had participated in an anterior cruciate ligament preventive training program.

Moreover, the majority of participants (55.28%) had taken an ACL injury PTP from coaches, and the rest (44.72%) had undergone the program with a physiotherapist (Figure 2).

A binary logistic regression test was used to demonstrate the association athletes in terms of their sport, high- and low-risk sports. A total of 1169 participants were included. When asked whether they knew what an ACL tear is, 829 participants answered yes. Moreover, 568 of them participated in high-risk sports, while 261 participated in low-risk sports (adjusted OR: 1.377; 95% CI: 1.009–1.879; *p*-value 0.044). A significant result was found regarding whether the participants knew a teammate who had sustained an ACL injury: 381 who played high-risk sports said yes, 65 said no (adjusted OR: 0.920; 95% CI: 0.663–1.274; *p*-value 0.614), and 309 answered that they did not know. Of the latter, 186 played high-risk sports, and 123 played low-risk sports (adjusted OR: 0.296; 95% CI: 0.199–0.441; *p*-value 0.000). A total of 985 participants, 545 from high-risk sports and 249 from low-risk sports, believed that ACL injuries are preventable (adjusted OR: 0.961; 95% CI: 0.393–2.351; *p*-value 0.930). Of those who said they did not know, 253 played high-risk sports and 91 played low-risk sports (adjusted OR: 2.159; 95% CI: 1.549–3.008; *p*-value 0.000).

When asked whether they were familiar with the concept of ACL injury PTPs, the number of participants who said no (971) was significantly higher than that of those who said yes (198); of the latter, 167 played high-risk sports, and 31 played low-risk sports (adjusted OR: 2.106; 95% CI: 1.544–2.873; *p*-value 0.000). When asked whether they had participated in an ACL injury PTP, 1,001 participants responded no, and 168 responded yes (adjusted OR: 1.637; 95% CI: 1.052–2.546; *p*-value 0.029). Finally, regarding whether they would perform a daily exercise program if it could prevent ACL injury, 899 participants said yes (adjusted OR: 1.484; 95% CI: 1.106–1.990; *p*-value 0.008; Table 4).

## 3. Discussion

We found that 63.1% of the study participants knew what an ACL injury is. A significantly higher proportion of these participants engaged in high-risk sports compared to those who did not know or engaged in low-risk sports (*p* = 0.044), as shown in Table 4. Compared to another study held in Hail between 2019 and 2020, the percentage of knowledge about ACL injury was 79.8% in our study. We believe the higher knowledge among the Hail population was due to the small sample size and the majority of study participants having a high educational level (University, Diploma and higher) [17]. In addition, a 2017 study conducted in Al-Bahah in Saudi Arabia concluded that the level of knowledge of ACL injury reached up to 77.8% [18]. Another study conducted in Saudi Arabia in 2020 observed that the percentage of knowledge about ACL injury was only 56% [19].

The percentage of participants who believed that female athletes are at higher risk of developing ACL injury was 19.8%. The previously mentioned 2020 study conducted in Saudi Arabia found that the percentage of those who believed this was 14.6% [19]. A study conducted in the United States found that the percentage of athletes who believed this was 85% [16]. The low percentage in the current study may have been due to the low numbers of women practicing sports in Saudi Arabia over the past few years, affecting the level of awareness of their sports-related injuries.

In this study, 40.9% of participants believed that female and male athletes should receive the same treatment for ACL injury. Meanwhile, a study conducted in the United States found that the percentage of athletes who believed this was 35% [16]. Moreover, in the current study, 67.2% of participants believed that ACL injuries can be prevented. A study conducted in the United States in 2017–2018 found a slightly higher percentage (89%) of American athletes believed this [20]. The reason for the lower percentage in the current study may have been due to Saudi athletes’ lack of awareness of programs concerning ACL injury and its prevention.

In this study, the athletes who practiced high-risk sports, such as football, basketball, and volleyball, had a better level of information about ACL injury than those who practiced lower-risk sports, such as cardio. These results were consistent with those of a study conducted in the United States where athletes practicing high-risk sports had a higher level of knowledge of ACL injury than those practicing less extreme sports [16]. In the current study, more of the high-risk sport participants knew a team member who had had an ACL injury, as compared to those who practiced lower-risk sports, consistent with the results of the previously mentioned study conducted in the United States [16]. Those who practiced high-risk sports in this study believed that they could better prevent ACL injuries compared to those who practiced lower-risk sports, which was consistent with the results of another study conducted in the United States [16]. Several studies have confirmed that it is possible to prevent ACL injury through PTPs [20,21,22], and the current study found that the high-risk sport participants were more aware of the concept of PTP for ACL injuries, consistent with the results of the aforementioned study conducted in the United States [16].

The current study found that more participants who practiced high-risk sports had undertaken a PTP due to ACL injury than those who practiced low-risk sports, which was similar to the results of the other study. Additionally, more high-risk sport participants answered that they would take part in a daily exercise program if it could prevent ACL injury, as compared to those who practiced lower-risk sports, consistent with the results of the aforementioned study conducted in the United States [16].

The most common source of information about ACL injuries among the current study’s participants was sport coaches at 70.97%, followed by the athlete’s university at 10.37%, and the internet at 7.60%. However, a 2020 study conducted in Saudi Arabia found that the most common information source was the internet at 27.5%, followed by television at 22.3%, and sport trainers at 6% [4]. In addition to another study that was held in Hail in 2019–2020, the most common source of information about ACL injuries was the internet, followed by television [17]. The reason for this discrepancy may have been that the other two studies were aimed at the general public, while the current study was directed at athletes in particular, which could have been an influencing factor on the source of ACL injury information.

Finally, the study participants answered that 55.28% of the professionals who headed the PTPs were sport trainers, followed by physical therapists at 44.72%. Similarly, in the previously mentioned study conducted in the United States, the majority of PTP presenters were trainers, followed by physical therapists [16].

## 4. Limitations

This study had several limitations. The tool used was a self-report survey. Therefore, it was not possible to verify the accuracy of the survey responses, especially as they related to the demographic data, such as age, gender, and affiliation, of the participants eligible for inclusion. Given the lack of studies on this topic, more research is needed.

## 5. Conclusions

In general, the awareness level of ACL injury PTPs among the Saudi athletes surveyed was poor. This indicates that medical professionals must increase efforts to improve such awareness. In public settings, health education programs about ACL preventive training and the importance of the condition should be implemented. Moreover, primary care physicians should be trained and supervised to raise community awareness. More studies are needed to assess the degree of social awareness, which is the most effective way to reduce or eliminate untreated ACL injuries.

## Figures and Tables

**Table 1:** The 12-item survey sent to current Female National Collegiate Athletic Association athletes.

No	Question
1	Do you know what an anterior cruciate ligament (ACL) injury is?
2	Have you ever sustained an ACL injury?
3	Do you know of any teammates who have sustained an ACL injury?
4	(True/False) Female athletes are at increased risk for sustaining ACL injuries than male athletes.
5	(True/False) Female and male athletes should have the same treatment for the same injuries.
6	(True/False) ACL injuries can be preventable.
7	Have you ever been educated on ACL injuries?
7a	If yes, by whom? (coach, athletic trainer, other: _ write N/A)
8	Are you familiar with the concept of an ACL preventive training program?
8a	If yes, where did you hear about this? (coach, athletic trainer, other:_)
9	Have you ever performed an ACL preventive training program?
9a	If yes, who oversaw this? (coach, athletic trainer, PT, parent, self, other: _
10	Do you currently perform an ACL preventive training program?
11	Would you perform a daily exercise program if you knew it could prevent ACL injury?
12	what is your level of interest in learning more about ACL preventive training programs?

**Table 2:** Baseline sociodemographic characteristics of the study participants (n: 1169).

Variable	Frequency	Percentage
Age, years (mean: SD)		
30.99 ± 11.25		
Gender		
Male	561	48%
Female	608	52%
Place of residence		
Central region	246	21%
Western region	314	28.9%
North region	216	18.5%
Eastern region	165	14.1%
South region	228	19.5%
Sport		
football	428	36.6%
Cardio	255	21.8%
Basketball	64	5.5%
Volleyball	49	4.2%
Handball	20	1.7%
Athletics	125	10.7%
Water sports	109	9.3%
Bicycle	85	7.3%
Tennis	32	2.7%
Boxing	2	0.2%

SD: standard deviation.

**Table 3:** Survey responses of Saudi athletic association athletes concerning their understanding of anterior cruciate ligament injuries (n = 1169).

**Variable**	**Frequency**	**Percentage**
Know what an anterior cruciate ligament (ACL) tear is		
Yes	738	63.1%
No	431	36.1%
Believe female athletes are at increase risk of sustaining ACL injuries		
Yes	238	18.8%
No	238	20.4%
Don’t know	700	59.9%
Believe female and male athletes should have the same treatment for the same injuries		
Yes	478%	40.9%
No	172%	14.7%
Don’t know	519%	44.4%
Believe ACL injuries are preventable		
Yes	785	67.2%
No	40	3.4%
Don’t know	344	29.4%
Have been educated on ACL injuries		
Yes	434	37.1%
No	735	62.9%
what is your level of interest in learning more about ACL preventive training programs?		
I am not interested	104	8.9%
Interested, but I don’t expect any benefit from it	44	3.8%
neutral	91	7.8%
Slightly interested	203	17.4%
Moderately interested	189	16.2%
Somewhat interested	113	9.7%
Greatly interested	426	36.4%

ACL: Anterior cruciate ligament.

**Table 4:** Binary logistic regression used for the adjusted analysis and determining association athletes in terms of sport played, high- and low-risk sports.

Predictor Variables	Risk Possibility		aOR	95% Confidence Interval	*p*-Value
	High Risk	Low Risk			
Know what an anterior cruciate ligament (ACL) tear is					
Yes	568	261	1.377	1.009-1.879	0.044 *
No	170	170	1	Ref.	
Have sustained an ACL injury					
Yes	109	18	1.469	1.009–2.838	0.252
No	720	322	1	Ref.	
Know a teammate who sustained an ACL injury					
Yes	381	65	0.920	0.663–1.274	0.614
No	262	152	1	Ref.	
Don’t know	186	123	0.296	0.199–0.441	0.000 *
Believe female athletes are at increased risk of sustaining ACL injuries					
Yes	177	54	0.778	0.520–1.164	0.221
No	180	58	1	Ref.	
Don’t know	472	228	0.944	0.633–1.407	0.776
Believe ACL injuries are preventable					
Yes	545	240	0.961	0.393–2.351	0.930
No	31	9	1	Ref.	
Don’t know	253	91	2.159	1.549–3.008	0.000 *
Familiar with the concept of an ACL injury preventive training program (PTP)					
Yes	167	31	2.106	1.544–2.873	0.000 *
No	662	309	1	Ref.	
Have performed an ACL injury PTP					
Yes	148	20	1.637	1.052–2.546	0.029 *
No	681	320	1	Ref.	
Would perform a daily exercise program if it could prevent ACL injury					
Yes	658	241	1.484	1.106–1.990	0.008 *
No	171	99	1	Ref.	

High risk: Football, basketball, volleyball, handball, athletics, water sports, tennis, boxing; Low risk: Cardio, cycling; aOR: Odds Ratio; PTP: Preventive training programs; * Alpha criterion was set to 0.05 or less to be considered a significant value.

## Data Availability

The data presented in this study are available on request from the corresponding author.
